# An Insight into Methods and Practices in Hip Arthroplasty in Patients with Rheumatoid Arthritis

**DOI:** 10.1155/2015/140143

**Published:** 2015-07-08

**Authors:** Mohammad Saeed Mosleh-shirazi, Mazin Ibrahim, Philip Pastides, Wasim Khan, Habib Rahman

**Affiliations:** ^1^Heart of England NHS Foundation Trust, Birmingham Heartlands Hospital, Bordesley Green East, Birmingham B9 5SS, UK; ^2^Royal Free London NHS Foundation Trust, London NW3 2QG, UK

## Abstract

Total hip arthroplasty (THA) has improved the quality of life of patients with hip arthritis. Orthopedic community is striving for excellence to improve surgical techniques and postoperative care. Despite these efforts, patients continue facing postoperative complications. In particular, patients with rheumatoid arthritis display a higher risk of certain complications such as dislocation, periprosthetic infection, and shorter prosthesis durability. In this review we present the current knowledge of hip arthroplasty in patients with rheumatoid arthritis with more insight into common practices and interventions directed at enhancing recovery of these patients and current shortfalls.

## 1. Introduction

Rheumatoid arthritis (RA) is a systemic autoimmune disease that affects 2% of women and 0.5% of men in the UK [1, 2]. This disease is characterized by peripheral and systematic polyarthritis, resulting in joint deformity and destruction due to erosion of cartilage and bone [3]. Rheumatoid patients requiring hip arthroplasty are usually younger than patients with osteoarthritis, display chronic systemic inflammation, use immune suppressive and disease-modifying drugs, and frequently develop osteoporosis [4–6].

The goal of treatment for patients suffering from rheumatoid arthritis of the hip is to reduce pain and improve function [7]. These patients are already taking analgesics, NSAID, and immunosuppressive and disease-modifying drugs. However, at advanced stages of the disease, surgical treatments become essential [7]. Once hip is involved in rheumatic disease process the function and mobility of these patients are greatly reduced and these patients go off their feet very quickly. Total hip arthroplasty (THA) is a surgical intervention carried out on end-stage arthritis patients.

According to the National Hip and Knee Registry, in 2011, in the UK, hip and knee replacements have increased by 5% and 3.3% compared to 2010. The necessity to assess the rate of developing complications in RA patients can be understood in light of implications it may have on surgical decision-making. In other words, understanding the risk factors involved and higher vulnerability of groups of patients with specific characteristics is a critical factor in determining which patients should receive surgical treatments.

In this review we have sought to specifically address causes of complications, benefits and risk involved in undertaking THA, measures aimed at enhancing recovery, and the improvement of quality of life in these patients. Furthermore we will debate common practices and shortfalls in surgical interventions, techniques, and postsurgical care.

We searched the literature using electronic medical databases including PUBMED, MEDLINE, and EMBASE up to August 2014 on reports pertaining to current knowledge of the field of total hip arthroplasty (THA) in patients suffering from rheumatoid arthritis (RA). We have used the following keywords: “Arthroplasty,” “Rheumatoid Arthritis,” “Total Hip Arthroplasty,” “Total hip replacement,” “Peri-operative management,” and “Post-operative management.”

RA patients undergoing THA treatment can encounter more complications as compared to patients suffering from osteoarthritis (Table 1). Here, we summarize causative factors that may be contributing to the observed complications and also discuss preventative measures and procedures that may minimize such risks.

## 2. Complications of THA in Patients with RA and Causative Factors Contributing to These Complications

Despite recent improvement in biological agents and treatment procedures in the field of rheumatology, joint and musculoskeletal deterioration continues to occur in patients with RA, who eventually require joint surgery. In patients with total hip arthroplasty, any of the following complications may be experienced: hip dislocation, requirement of early revision surgery, shorter prosthesis durability, joint infection, venous thromboembolism, and death [8]. Recent reports suggest that patients with RA may be at a greater risk of developing some of these complications.

In a large cohort study conducted by Ravi and colleagues, in two age- and sex-standardized groups of patients with OA and RA both undergoing THA, the latter group was at a twofold higher risk of dislocation compared to OA patients [8]. In addition, this study showed that RA patients were more likely to develop surgical complications but less likely to develop venous thromboembolism. Put together, these studies suggest that RA can be a major risk factor in patients undergoing hip arthroplasty.

In regard to trends of THA rates, a recent population-based study in the US has revealed that THA rates have minimally decreased amongst patients with rheumatoid arthritis [9].

In this section, we will elaborate on causative factors contributing to higher susceptibility of RA patients for each complication.

### 2.1. Dislocation in RA Patients

Dislocation is one of the most encountered complications following THA [10, 11]. Earlier studies have suggested that patients with RA who undergo joint arthroplasty are at a significantly higher risk of dislocation as compared to patients with osteoarthritis [12–15]. Despite the controversy regarding rates of dislocation, we have sought to focus on a number of underlying factors contributing to dislocation specifically in RA patients.

Firstly, the implant head sizes used for patients with RA are usually smaller than OA patients due to the lower average BMI in patients with OA. The tendency to use prosthesis with smaller head sizes increases the risk of dislocation [16]. For instance, Khatod et al. report a 3-fold higher risk of dislocation with 28 mm head sizes as opposed to 32 mm head sizes in THR patients [16]. The elevated risk of dislocation in RA patients compared to OA patients may be a result of pathological anatomic differences. Specifically, protrusio acetabuli is more common in RA patients compared to OA patients. In RA patients, the upward migration of the acetabular roof, collapse of the femoral head, and destruction of the acetabulum may lead to dislocation. It is thought that the use of adrenal cortical steroids destruction may be the underling etiology [17, 18]. In addition RA patients have a higher tendency to develop osteoporosis [19] and mainly show worse bone quality than OA patients [7].

The increased risk of dislocation may be due to soft tissue laxity compared to OA patients [20]. The studies aimed at investigating the cause for the elevated risk of dislocation in RA patients indicate that factors such as surgical factors (soft tissue laxity), mechanical factors (less hip abductor strength and protrusion acetabuli), RA disease state, and inflammation of soft tissues may be contributory. Several studies have recommended using constrained acetabular component to prevent dislocation in patients at an elevated risk of dislocation [21–24].

Based on these reports we can conclude that RA remains an important risk factor for dislocation following THA. The relatively worse quality of bone and soft tissues in these patients compared to OA patients is likely to be the cause of more frequent dislocations observed. The use of a constrained acetabular component is an effective option for preventing dislocations in THA in high-risk patients. This option may be elected for patients with RA undergoing THA.

### 2.2. Periprosthetic Infection

Periprosthetic joint infection is one of the most dreaded complications after THA. The association between increased risks of infection in RA patients undergoing THA has been frequently studied [25]. There have been reports of increased risk of infection in patients with RA compared with patients with OA [26], possibly due to the systemic autoimmune disorder. Schrama and colleagues compared the differences in the risk for revision due to infection in RA and OA patients undergoing THR. They found that RA patients did not have higher risk of early infection. However, these patients have a higher risk of late infection, leading to revisions [27]. Bongartz and colleagues also report an increased risk of periprosthetic joint infection in RA patients [28].

In contrast to these studies, surprisingly, a study conducted in Brazil by da Cunha and colleagues indicated that no elevated risk of infections was detected between the OA and RA patient groups [3].

In studies aimed at investigating the cause for the elevated risk of infection in RA patients, factors such as RA disease state, inflammation, and the choice of medication (use of disease-modifying antirheumatic drugs (DMARDs)) were linked to the higher risk of infection [29, 30]. In regard to the choice of medications, nonbiologic disease-modifying antirheumatic drugs (DMARDs), including methotrexate, or low dose corticosteroids may not increase the risk of infection in RA [31]. By contrast, biologic disease-modifying antirheumatic drugs (biologic DMARDs), such as infliximab and etanercept, have been associated with a higher risk of prosthetic joint infection [29].

### 2.3. Shorter Prosthesis Durability and Choice of Implant

Choice of implant fixation in THA remains controversial [3, 13, 18, 21], particularly in patients with RA [32, 33]. Earlier studies did not present any evidence of shorter prosthesis durability in patients with RA [34, 35]. In RA patients, cemented prosthesis is thought to function inferiorly when compared to osteoarthritis patients [36–38]; however, this general consensus has been challenged by other studies, reporting no detectable difference [7, 39]. The use of noncemented prosthesis, though questionable in these patients, has been promising [40, 41]. In a study conducted by Ejnisman and colleagues, the use of uncemented THA implants in patients with RA and OA was compared. No significant difference was found in osseointegration rates in either group. Furthermore, the rate of requirement of revision surgery and implant survival was not significantly different. The use of uncemented HA-coated prosthesis [42] and porous prosthesis [33], in short-term studies, has generated promising results [43]. In more recent years, the use of porous-coated stems and cemented all-polyethylene cups has been recommended as implants of choice in young patients with RA [44].

In a comparative study conducted in 2011, three patient groups were studied: cementless group (a noncemented porous-coated stem and a noncemented porous-coated cup), cemented group 1 (a cemented, loaded-taper stem combined with a cemented, all-polyethylene cup), and cemented group 2 (a cemented, composite-beam stem with a cemented all-polyethylene cup). The authors concluded that cementless stems and cups had a significantly lower risk of revision for aseptic loosening than cemented implants in patients with RA [45]. In conclusion, the new generation porous-coated cementless stems and cups may be the implant of choice in patients with RA; however more research is needed in this field.

In addition to the implant of choice, the longevity of THA in RA patients is also dependent on bone quality supporting the prosthesis. Bone remodeling is a major characteristic of these patients; therefore, the bone-cement bonding may be compromised in RA patients as a consequence of their disease. [43]. Even though RA patients are mainly younger than patients with osteoarthritis, RA patients have poor bone stock and compromised musculoskeletal structure; therefore, a higher risk of shorter prosthesis durability can be expected in these patients [43]. However, recent studies have challenged these reports and have not found a significant difference in the survival of the cups between THAs in RA and OA patients [46]. Therefore, in addition to type of implant, the age and bone quality of the RA patient also impact the longevity of THA.

## 3. Current THA Practices and Areas for Improvement in Patients with RA

### 3.1. Perioperative Medications

In RA patients compared to OA patients, undergoing THA, the perioperative risk of complications and the requirement to use health care resources continue to rise [47]. Despite the benefits of THA surgery for RA patients, the risk of perioperative infection is a risk that these patients face. These patients require meticulous preoperative assessment of their infection risks. One strategy to decrease the risk of infection and enhance healing is to modify medication regimens [48].

The use of rigorous preventive measure such as intraoperative antisepsis, postoperative antimicrobial prophylaxis can reduce the incidence of prosthetic joint infection and overall costs [28]. The codes of practice for reduction of surgical infection do not necessarily differ greatly between rheumatologic and general patient populations but, ultimately, the best practice relies on correct assessment and implementing adjustments in caring for patients with this illness [48].

The use of biological agents has been associated with a higher risk of infection in RA patients [29]. For instance, a recent study recommended performing arthroplasty before introduction of TNF-a blockers. They also recommended withdrawal of TNF-a blockers during perioperative period and reduction of steroid intake as low as possible [49].

The American College of Rheumatology in 2008 issued recommendations stating that biological agents should be withheld at least 1 week before and 1 week after arthroplasty in patients with RA [50]. However, several aspects of this recommendation and its implications were unexplored. Firstly, the benefit versus risks of withholding medications for RA patients, which may be reducing the risk of infection versus RA flaring, was not discussed. Secondly, no recommendations were made regarding withholding traditional DMARDs such as methotrexate; therefore no solid conclusion can be drawn and finally in the absence of statistical evidence and clinical data, the validity and applicability of these recommendations remain unclear [51]. The use of these drugs before and after THA surgery requires a significant level of clinical study and follow-up, so that safe conclusions can be drawn.

### 3.2. Patient Education

The elevated risk of prosthesis dislocation, prosthetic joint infection, and shorter prosthesis durability in RA patients compared to OA patients should be made known to patients elected for surgical option by healthcare providers such as rheumatologists and orthopaedic surgeons. This information should be provided as informed consent. To that end, policy makers should also incorporate this information in their assessment protocols of patients undergoing THA [51]. In a study conducted on patients undergoing THA and TKA, a one-on-one preoperative educational program resulted in significantly shorter length of stay [52] suggesting that patient awareness can play an important role in enhancing recovery. Engaging patients in preoperative patient education and rehabilitation should in principle decrease their length of stay at the hospital.

### 3.3. Peri- and Postoperative Management and Methods of Rehabilitation

Ideally, perioperative medical evaluation for rheumatological cases undergoing orthopaedic surgery should initiate several weeks before elective surgery [53].

RA patients undergoing THA have a higher risk of complications as compared to non-RA patients. Therefore, the correct preoperative medical assessment and initiation of preventive strategies could massively benefit patients [28]. Specifically, specialised perioperative care and systematic management in regard to their immunosuppressant medication, evaluation of their cardiovascular state, and cervical spine disease are highly recommended [12].

In regard to postoperative care, RA patients require special attention because of their disease state and treatments. In regard to risk factors after THA surgery, the use of TNF-*α* and prednisolone was identified as risk factors for MRSA infection [49]. Given this example, specific evaluation of an individual patient, assessment of risk factors associated with the patient, and ensuring systematic peri- and postoperative management of this patient could directly decrease possible complications.

## 4. Recommendations for Further Improvements

It is evident that taking up a patient with RA for consideration of hip arthroplasty requires heightened pre-, peri-, and postoperative considerations.

Optimization of preoperative medical management is essential and involvement of rheumatologist is always recommended.

Implant selection appears pivotal in the outcome. Cementless prosthesis in the right hands has yielded the best outcome thus far.

Meticulous surgical techniques are always paramount in any implant insertion surgery and more so in case of RA patients to reduce infection risks.

## Figures and Tables

**Table 1:** Table showing rate of complications in RA patients undergoing THA.

Author	Total THA	Implant	RA	Revisions	Dislocations	Infection	Other
Ravi et al. [8]	60,305		1,163 (3%)		596 (1.3%)	487 (1.1%)	60 (0.4%) fractures

McCollum and Gray [10]	441				1.14%		

Conroy et al. [11]	58109			428 (0.7%)			

Khatod et al. [16]	1693			277 (16%)	28 (1.7%)		

Mibe et al. [18]	19			2 (10%)			Death (2%)

Bigsby et al. [21]	117	Omnifit		1 (0.8%)			Death (45.3%) 3 y

Jones et al. [23]	81	Acetabular			3 loosenings (3.7%)		

Shrader et al. [24]	20				10-cup loosenings (50%) 6-stem loosenings (30%)		

Schrama et al. [27]			4,167	25 (0.6%)			99.9% 1 y survival

Bongartz et al. [28]			462			23 (3.7%)	

Momohara et al. [29]			81			3 (0.7%)	

Lacaille et al. [31]			27,710			Mild 25,608 (92%) Serious 4,941 (18%)	

Himanen et al. [34]			2,161				97% 5 y survival 96% 10 y survival

Tang and Chiu [43]			28	1 revision	2 loosenings	2 deep infections	

Eskelinen et al. [44]			2,557				89% 15 y survival

Stundner et al. [47]	157,775		5400 (3.42%)				Younger/more likely female

Gilson et al. [49]			18			5 (25%)	
