# Corrigendum to “An Insight into Methods and Practices in Hip Arthroplasty in Patients with Rheumatoid Arthritis”

**DOI:** 10.1155/2016/1741420

**Published:** 2016-12-27

**Authors:** Mohammad Saeed Mosleh-shirazi, Mazin Ibrahim, Philip Pastides, Wasim Khan, Habib Rahman, Leila Jahangiri

**Affiliations:** ^1^Heart of England NHS Foundation Trust, Birmingham Heartlands Hospital, Bordesley Green East, Birmingham B9 5SS, UK; ^2^Royal Free London NHS Foundation Trust, London NW3 2QG, UK; ^3^Ludwig Institute for Cancer Research, University of Oxford, Oxford, UK

In the article titled “An Insight into Methods and Practices in Hip Arthroplasty in Patients with Rheumatoid Arthritis” [1], Dr. Leila Jahangiri was missing from the authors' list. Dr. Jahangiri contributed to the writing and revision of the article. The corrected authors' list is shown above.
